# The effect of high-definition transcranial direct current stimulation intensity on motor performance in healthy adults: a randomized controlled trial

**DOI:** 10.1186/s12984-021-00899-z

**Published:** 2021-06-26

**Authors:** Ohad Lerner, Jason Friedman, Silvi Frenkel-Toledo

**Affiliations:** 1 Department of Physical Therapy, Faculty of Health Sciences, Ariel University, Ariel, Israel; 2 Department of Physical Therapy, Stanley Steyer School of Health Professions, Sackler Faculty of Medicine, Tel Aviv University, Tel Aviv, Israel; 3 Sagol School of Neuroscience, Tel Aviv University, Tel Aviv, Israel; 4 Department of Neurological Rehabilitation, Loewenstein Hospital, Raanana, Israel

**Keywords:** High-definition transcranial direct current stimulation, Current intensity, Motor performance

## Abstract

**Background:**

The results of transcranial direct current stimulation (tDCS) studies that seek to improve motor performance for people with neurological disorders, by targeting the primary motor cortex, have been inconsistent. One possible reason, among others, for this inconsistency, is that very little is known about the optimal protocols for enhancing motor performance in healthy individuals. The best way to optimize stimulation protocols for enhancing tDCS effects on motor performance by means of current intensity modulation has not yet been determined. We aimed to determine the effect of current intensity on motor performance using–for the first time–a montage optimized for maximal focal stimulation via anodal high-definition tDCS (HD-tDCS) on the right primary motor cortex in healthy subjects.

**Methods:**

Sixty participants randomly received 20-min HD-tDCS at 1.5, 2 mA, or sham stimulation. Participants’ reaching performance with the left hand on a tablet was tested before, during, and immediately following stimulation, and retested after 24 h.

**Results:**

In the current montage of HD-tDCS, movement time did not differ between groups in each timepoint. However, only after HD-tDCS at 1.5 mA did movement time improve at posttest as compared to pretest. This reduction in movement time from pretest to posttest was significantly greater compared to HD-tDCS 2 mA. Following HD-tDCS at 1.5 mA and sham HD-tDCS, but not 2 mA, movement time improved at retest compared to pretest, and at posttest and retest compared to the movement time during stimulation. In HD-tDCS at 2 mA, the negligible reduction in movement time from the course of stimulation to posttest was significantly lower compared to sham HD-tDCS. Across all groups, reaction time improved in retest compared to pretest and to the reaction time during stimulation, and did not differ between groups in each timepoint.

**Conclusions:**

It appears that 2 mA in this particular experimental setup inhibited the learning effects. These results suggest that excitatory effects induced by anodal stimulation do not hold for every stimulation intensity, information that should be taken into consideration when translating tDCS use from the realm of research into more optimal neurorehabilitation.

*Trial registration:* Clinical Trials Gov, NCT04577768. Registered 6 October 2019 -Retrospectively registered, https://register.clinicaltrials.gov/prs/app/action/SelectProtocol?sid=S000A9B3&selectaction=Edit&uid=U0005AKF&ts=8&cx=buucf0.

**Supplementary Information:**

The online version contains supplementary material available at 10.1186/s12984-021-00899-z.

## Introduction

A major goal of clinical neuroscience is to develop effective, non-invasive methods for improving function via neuroplasticity modulation. One non-invasive and painless stimulation method that has received increasing attention is transcranial direct current stimulation (tDCS). It delivers weak direct currents (usually 0.5–2 mA) through surface electrodes placed on the skull. The current does not directly induce cerebral activity, but rather alters spontaneous brain activity and excitability by the subthreshold modulation of neuronal membranes in a polarity dependent manner [for a review see [1]]. It is commonly assumed that anodal stimulation leads to a subthreshold depolarization and increased cortical excitability whereas cathodal stimulation leads to hyperpolarization and decreased cortical excitability [for a review see [1, 2]]. tDCS has been shown in some studies to be an effective means to improve motor performance in healthy subjects as well as patients suffering from neurological diseases such as stroke and Parkinson’s disease [for reviews see [3–6]]. Yet, the results of clinical studies, such as those aimed at improving motor recovery following stroke by targeting the primary motor cortex (M1), have been inconsistent [for reviews see [7, 8]], probably because, among other reasons, very little is known about the optimal protocols for enhancing motor ability in healthy individuals.

Modulating stimulation intensity may optimize stimulation protocols to enhance the effects of tDCS on motor performance. The examination of neurons of animal brains has found no conclusive evidence for a linear dose–response relationship at electric field intensities below 1 V/m; however, there is evidence of neurophysiological changes at specific low intensities [for a review see [9]]. In humans, the effects of stimulation intensity on neurophysiological and behavioural measures are inconsistent. From a neurophysiological point of view, an early study indicated that stronger anodal stimulation, delivered in the range of 0.2 to 1 mA, to the left motor cortex using large surface sponge electrodes tended to induce greater Motor Evoked Potentials (MEP), a commonly used measure of cortical excitability [2]. Similarly, Ammann et al. [10] found that the higher stimulation intensity of 2 mA, but not 1 mA, applied via conventional large pad anodal tDCS (atDCS) delivered for 7 min over the primary motor cortex significantly increased cortical excitability. On the other hand, Bastani and Jaberzadeh [11] found that conventional large pad atDCS applied to the left primary cortex for 10 min with stimulation intensity of 0.3 mA induced significantly larger corticospinal excitability changes than 0.7 mA. There were no significant differences between the excitability changes for the 0.3 and 1.4 mA or 0.3 and 2 mA intensities. Additional studies found no significant differences in motor cortex excitability between stimulation intensities [for a review see [9, 12–17]]. For example, a systematic evaluation of the effect on motor cortical excitability of four intensities–0.5, 1, 1.5, and 2 mA–applied with conventional large pad atDCS to the left primary cortex for 15 min–found no significant differences on MEP after-effects [16]. In addition, higher intensities of conventional large pad atDCS to the left primary cortex—1, 2, and 3 mA—applied for 15–30 min induced similar MEPs [12]. Interestingly, some results even suggest that increasing the current intensity changes the direction of MEP after-effects [13, 18, 19].

From a behavioral point of view, to the best of our knowledge, the effect of stimulation intensity was investigated in only a few studies in different domains [20–25]. Using conventional large pad atDCS stimulation to the left prefrontal cortex for 20 min, 2 mA, but not 1 mA, improved verbal fluency in healthy subjects [22] and working memory in patients with Parkinson's disease [20]. Using smaller gel electrodes with high-definition (HD)-tDCS (4 × 1 ring electrode configurations) to left temporoparietal area and dorsolateral prefrontal cortex for 10 or 20 min, 2 mA caused a greater reduction in both tinnitus loudness and annoyance than 1 mA in participants with chronic tinnitus [24]. In the motor domain, a combination of motor learning and 1.5 mA atDCS, applied with conventional large pad over M1 contralateral to the hand performing the motor task for 20 min, led to a significant improvement of motor performance compared to sham stimulation in healthy subjects [21]. However, no significant differences were reported between 1.5 mA atDCS and 1 mA atDCS or between 1 mA atDCS and sham tDCS. Similarly, no significant effects of tDCS on measures of simple visual motor reaction time were found following 1 and 2 mA atDCS or cathodal tDCS [26]. In another study, an exhibited conditioned learning (delay eye blink conditioning behaviour) over time was found after 20 min of conventional tDCS of the cerebellum both at 1.5 mA and 2 mA [23]. In an inverted U-shaped dose–response curve found for a neurocognitive task, a moderate stimulus intensity (1 mA) had the strongest effect on performance (4 sessions of 0.7, 1 and 2 mA atDCS for 20 min) [25]. Additional file 1: Table S1 describes the details of the above studies, while relating to the effects of tDCS on both the neurophysiological and behavioral measures.

Most of the neurophysiological and behavioural research, including studies dealing with the effects of stimulation intensity, used conventional large pad tDCS, which delivers current to diverse brain regions, rather than only to the targeted region of interest. Thus, dose–response reflects the amalgamation of current flow across many regions with varied intensity in brain areas [9]. Improved spatial focality of tDCS can be achieved using HD-tDCS [27–30]. In comparison to conventional large pad tDCS, HD-tDCS (4 × 1 ring electrode configurations) demonstrated a peak induced electric field magnitude at the sulcus and adjacent gyri directly underneath the active electrode [29]. Therefore, using HD-tDCS, which enables a more nuanced control of current flow, may be more beneficial for determining dose–response related to the stimulation of a region of interest (although it does not altogether eliminate the confound of current spatial distribution).

This study is the first attempt to determine the effect of current intensity on motor performance using HD-tDCS, with optimized electrode configurations for maximal focal stimulation to the primary motor cortex, in healthy subjects. We chose to compare the 2 mA and 1.5 mA groups, and did not include a 1 mA group based on the findings of two previous studies that investigated the effects of tDCS intensities on motor tasks [21, 26]. A study that compared 1.5 mA with 1 mA tDCS showed that the motor performance that considers both the speed and accuracy of a finger sequence significantly improved at retention for 1.5 mA atDCS as compared to sham tDCS. No significant differences were reported between 1 mA atDCS and sham tDCS, or between 1.5 mA atDCS and 1 mA atDCS [21]. In contrast, in a study that compared 15 different simulation protocols atDCS–2 mA anodal, 2 mA cathodal, 1 mA anodal, 1 mA cathodal, or sham atDCS–across three different conditions (orbitofrontal, bilateral, or extracephalic reference electrode location), no significant effects of tDCS were found on simple motor reaction time [26]. In these studies [21, 26], the active electrode was placed over M1 and tDCS lasted for 20 min. Based on studies that investigated the effect of tDCS intensities [21, 26] and HD-tDCS [31, 32] on motor performance in healthy subjects, we hypothesized that the 1.5 mA HD-tDCS would be more effective in decreasing movement and reaction time than sham tDCS. Since, in the comparison between the effects of 1 mA, 1.5 mA and sham tDCS on motor performance, some positive effects were only found following 1.5 mA as compared to sham tDCS, and a trend towards positive effects was found following 1.5 mA as compared to 1 mA tDCS [21], and the comparison between 1 and 2 mA tDCS found no effects on motor performance at all [26], we also hypothesized that the 1.5 mA HD-tDCS would be more effective in decreasing movement and reaction time than 2 mA tDCS.

## Methods

### Study design

This was a single-blind, parallel, randomized, sham-controlled study. Data were collected in a brain and motor behavior laboratory based at Ariel University, Israel. Subjects were randomly assigned with a 1:1 ratio, using a random number generator in WINPEPI (by researcher SFT), to one of three groups: (1) HD-tDCS with an intensity of 2 mA (2 mA group); (2) HD- tDCS with an intensity of 1.5 mA (1.5 mA group); and (3) sham HD-tDCS (sham group). All participants were blinded to group allocation. To ensure blinding of participants, the stimulator monitor was hidden from the participants, and the sham stimulation increased and decreased in a ramp-like fashion (see HD-tDCS). The researcher (OL) who administered the HD- tDCS application and measured the outcomes received allocation information via coded email from another researcher (SFT). Blinding of group allocation was maintained during the data analysis. The trial was retrospectively registered at the ClinicalTrials.gov registry on October 6th, 2020 with the trial registration number NCT04577768.

### Participants

The sample size for this study was determined based on a power analysis calculation that was conducted using G*Power version 3.1.9.7. Power analysis yielded a total sample size of 54 individuals for the detection of a significant interaction with an assumed effect size of 0.25 and a power of 95%. To account for potential data loss, we aimed for a sample size of 20 individuals per group (in total 60 participants). Sixty subjects (34 women, 26 men; aged 25 ± 3 years) participated in the study between August 2019 to February 2020. Participants were included if they were aged between 20 and 35, were right-hand dominant and were healthy according to their report. They were excluded if they took psychiatric medications, had a history of drug abuse or dependence, had any psychiatric or neurological disorder, had a history of seizures, had metal implants in their head or had musculoskeletal deficits interfering with task performance (proper reaching performance in sitting). Participants signed an informed consent form prior to participating in the study. All the procedures were approved by Ariel University Institutional Review Board (approval number: AU-HEA-SFT-20190326), and were performed in accordance with relevant guidelines and regulations. Subjects were paid $20 for their participation.

### HD-tDCS

**Fig. 1:**
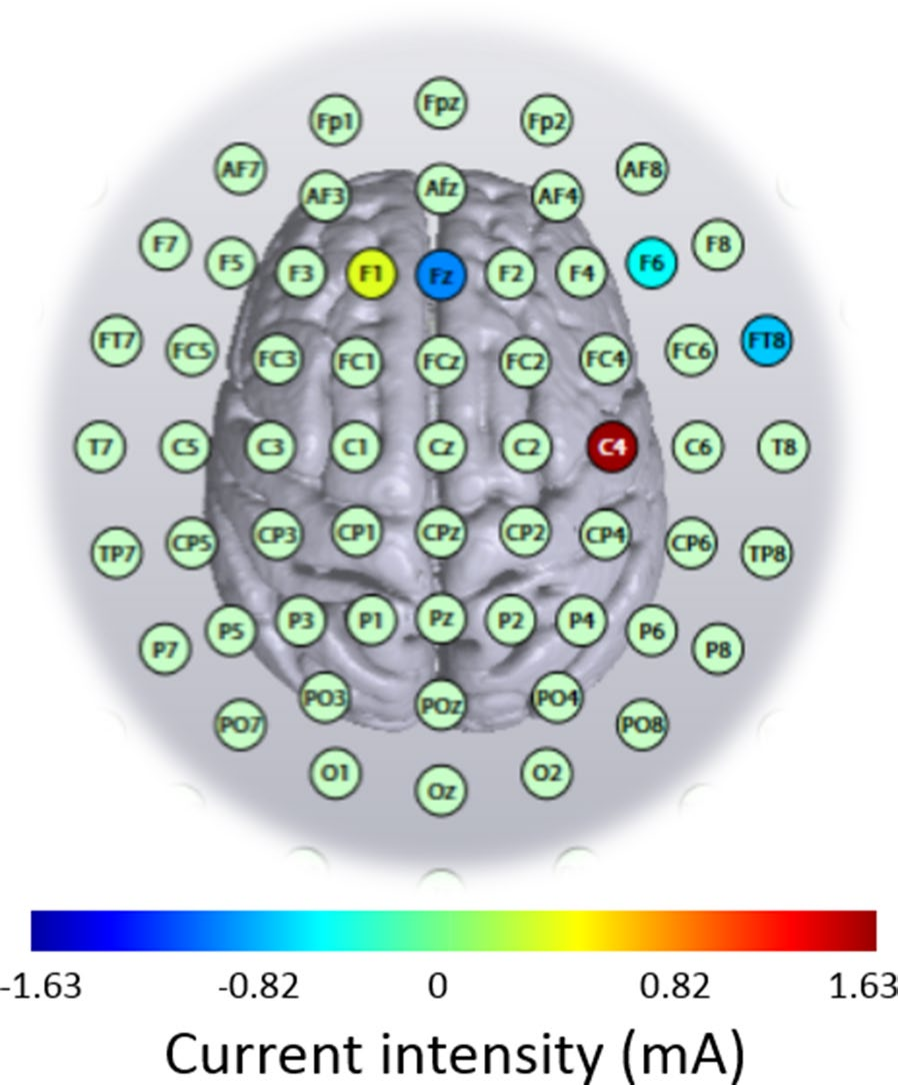
High-definition transcranial direct current stimulation (HD-tDCS) montage for maximal focal stimulation of the right Brodmann area 4 (primary motor cortex) using the HD-Targets modelling software (Soterix Medical, New York, NY). The location and current intensity value of each stimulating electrode are shown. Red denotes anodal stimulation while blue denotes cathodal stimulation

**Fig. 2:**
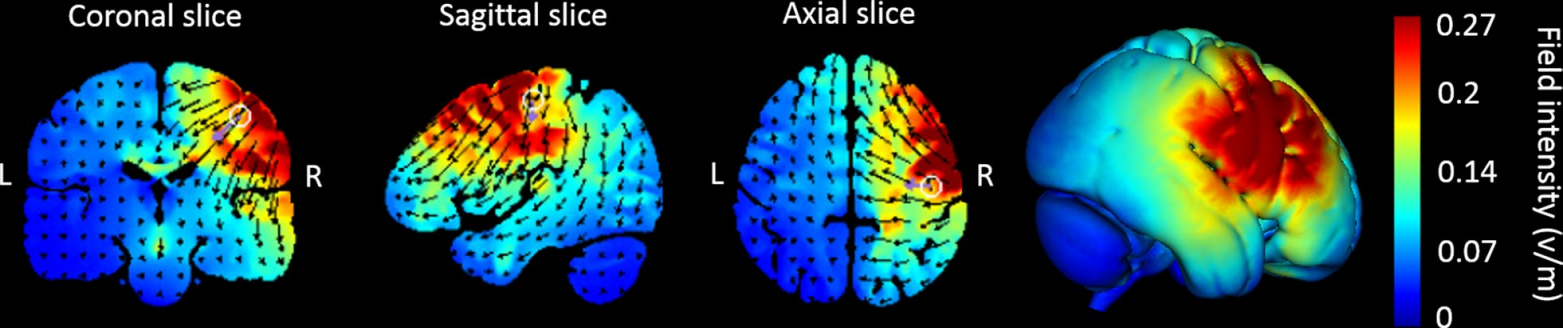
Current flow modeling during 2 mA High-definition transcranial direct current stimulation (HD-tDCS) using the HD-Target software (Soterix Medical, New York, NY). Current-flow models are shown on 2D and 3D reconstructions of the cortical surface. Skin, skull, and cerebrospinal fluid (CSF) masks are suppressed to reveal the underlying gray matter mask. The spatial profile of the current flow map is exactly the same as at 1.5 mA current injection but with induced electric field values scaled linearly. This is due to the linearity of the electric field solution [78]. A head model derived from the MNI 152 dataset was used

The stimulation was administered noninvasively using an M x N 9-channel high definition transcranial electrical current stimulator from Soterix Medical (New York, NY). Five sintered Ag/AgCl electrodes were attached to plastic holders, filled with conductive gel, and embedded in a HD cap, according to the extended 10–20 method of electrode placing. We administered a single session of 20 min of anodal stimulation targeting the right Brodmann area 4 (primary motor cortex; based on Talairach labels) by positioning electrodes at the following sites with the following intensities in the 2 mA and 1.5 mA HD-tDCS groups: C4 (1.63 mA and 1.22 mA, respectively), Fz (− 0.87 mA and − 0.65 mA, respectively), F1 (0.37 mA and 0.28 mA, respectively), F6 (− 0.47 mA and − 0.35 mA, respectively), and FT8 (− 0.66 mA and − 0.50 mA, respectively). HD-Targets brain modelling software (Soterix Medical, New York, NY) was used to determine the tDCS montage for maximal focal stimulation of the right primary motor cortex (Figs. 1 and 2). The HD-Targets brain modelling software replicates the procedures used by Dmochowski et al. [33], who showed that there are benefits for focality optimization. For example, at the intensity attained by the simulated sponge pad and 4-by-1 montage (0.16 V m ^− 1^) of a cortical target, the linearly constrained minimum variance optimization method (LCMV-ℓ1) yielded an 80% improvement in focality over the sponge pads and 47% over the 4-by-1 montage. In the 2 mA and 1.5 mA groups, the current increased in a ramp-like fashion over the course of the first 30 s, and decreased in a ramp-like fashion over the course of the last 30 s. In the sham group, once the current reached 2 mA over the first 30 s, it was ramped back down over 30 s. In the last min of the simulation an identical ramp up and ramp down occurred [for a similar approach see [34–37]]. Subjects were asked to report any adverse effects and to rank their discomfort from 1 to 10 following two min of stimulation.

### Motor sequence learning task

**Fig. 3:**
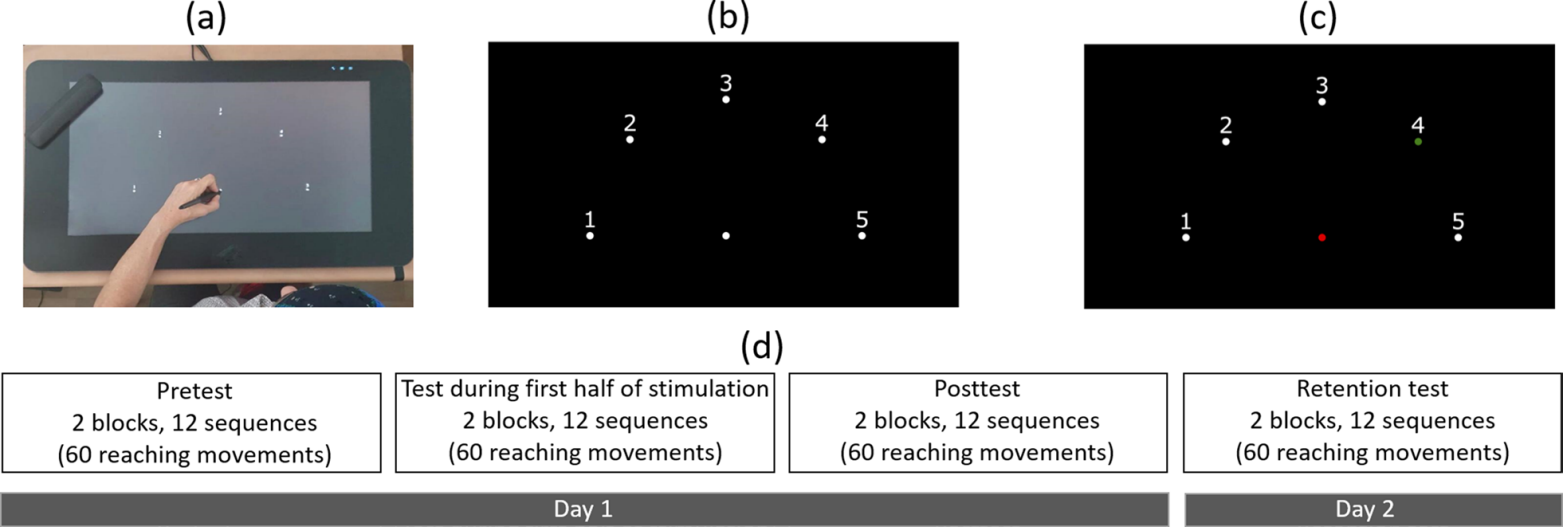
Experimental stimuli. (**a**) General setup of the motor task. (**b**) The participants started with the stylus at the starting point (the lower-middle target). After remaining there for 500 ms, (**c)** the center target turned red and one of the targets turned green, according to the current location in the sequence 4-1-3-2-5. After moving to and remaining at the target for 500 ms, the screen returned to (**b**), and the participants needed to lift the pen and return it to the starting point. Note that in this figure, for clarity, the targets and numbers are shown 3 times their relative size compared to those shown in the experiment. (**d**) Day 1 included pretest, test that started after two min of the appropriate stimulation and lasted six min, and posttest. Day 2 consisted of a retention test. Each of the tests (pretest, during stimulation, posttest and retention test) consisted of two blocks of 6 sequences (30 reaching movements within a block), with a 30 s break between blocks

In all subjects, the non-dominant left arm was tested. After placing the tDCS cap on the head, the subjects performed a sequential point-to-point movement task on the graphics tablet, a version of a similar, previously used task [e.g., [38–40]]. The stimuli consisted of a starting point, and five targets equally spaced around a semicircle, all equidistant from the starting point (17 cm), and all with a diameter of 0.5 cm (Fig. 3). Each movement began at the starting point. After holding the stylus at the starting point for 500 ms, the starting point changed color from white to red, and one of the targets changed color from white to green, after which the participants needed to move the stylus to the green target. They needed to remain there for 500 ms (until the target returned to its initial color), then lift the stylus and return it to the starting position to start the next movement. The participants were instructed that the targets would follow the sequence: 4-1-3-2-5, and to perform the task as fast and accurately as possible.

Initially, the participants were required to perform 3 sequences without errors to familiarize themselves with the setup, the task and the sequence. Then, they performed the pretest which consisted of two blocks of 6 sequences, i.e. 12 sequences, with a 30 s break between blocks. Two min after starting the appropriate stimulation, they performed 2 blocks of 6 sequences (identical to the pretest). After finishing the tDCS stimulation, the participants performed a post-test, which was also identical to the pretest. The participants returned after 24 h to perform a retention test, which was equivalent to the pre- and posttests.

Two outcome measures were used. The first was the movement time (s) of the reaching movements, defined as the time from movement onset (first time the tangential velocity was greater than 5% of the peak tangential velocity) until the end of the movement (the last time the tangential velocity was greater than 5% of the peak tangential velocity). The second measure was the reaction time (s), defined as the time between when the target appeared in green, and movement onset (as defined above). Improved motor performance was indicated by a shorter movement time and a shorter reaction time.

### Statistical analysis

Age and sex were compared between groups (2, 1.5 mA, sham) using Kruskal–Wallis (as age was not normally distributed) and chi-squared tests, respectively. The two outcome measures, movement time and reaction time, were normally distributed. The differences between groups with respect to each of the main outcomes in the pretest were investigated using one-way analysis of variance (ANOVA) with Bonferroni correction for multiple comparisons. The effects of stimulation and time on the outcome measures were investigated using a mixed design ANOVA with time (pretest, during stimulation, posttest, retest) as the within-subject factor and group (2, 1.5 mA, sham) as the between-subject factor with Bonferroni correction for multiple comparisons. The Greenhouse–Geisser Epsilon (G-GE) was used to correct the degrees of freedom when the Mauchly’s test of sphericity was significant. Change in movement time and reaction time was calculated by subtracting the movement time/reaction time values from a later time point to an earlier time point (that is, a negative value of change reflects improvement), and was compared between groups using one-way ANOVAs and post-hoc student`s t-tests with Bonferroni correction for multiple comparisons, when needed. The differences between groups with respect to the frequency of adverse effects was investigated using a chi-squared test. The differences between groups with respect to the discomfort from adverse effects was investigated using Kruskal–Wallis with Bonferroni correction for multiple comparisons. All tests were done using SPSS (version 26.0) with initial significance levels of *p* < 0.05.

## Results

**Fig. 4:**
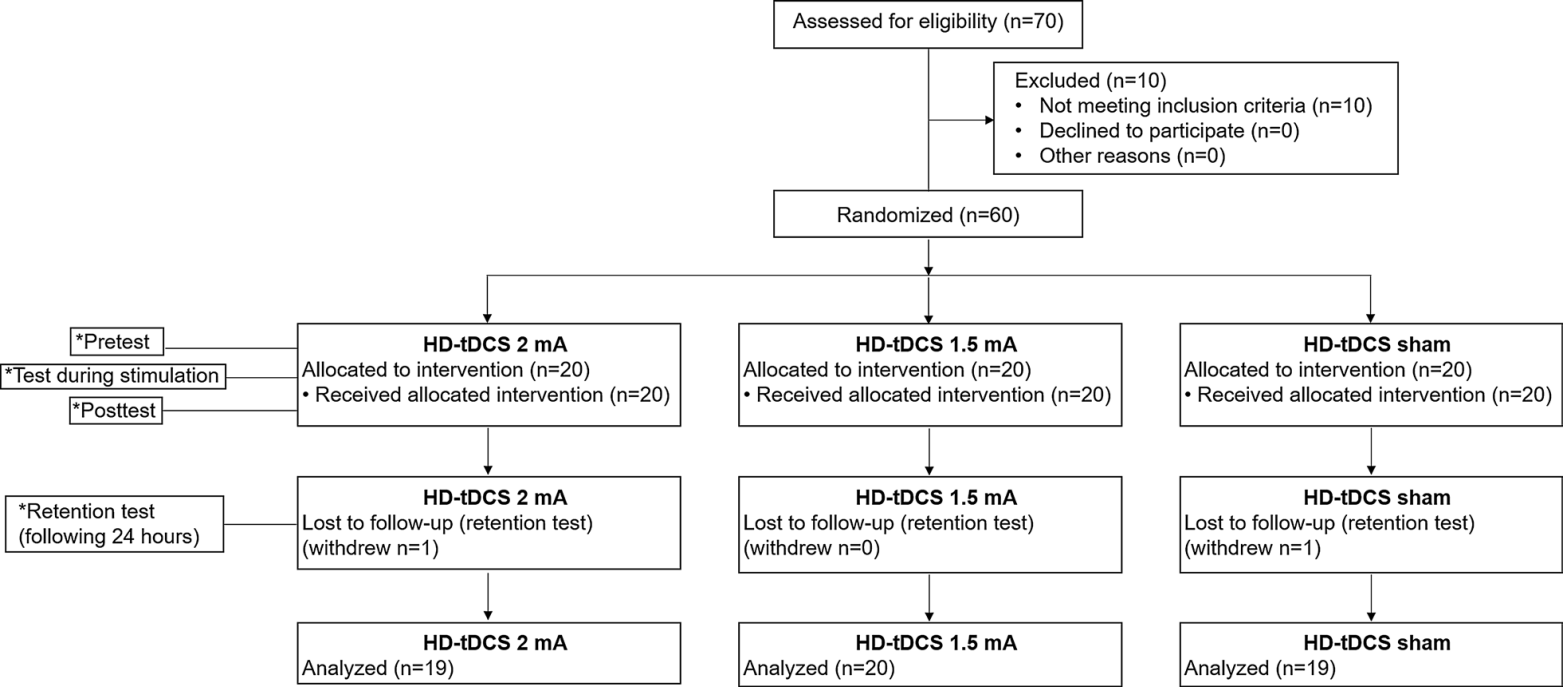
Trial flowchart. HD-tDCS 2 mA/1.5 mA = High-definition transcranial direct current stimulation with an intensity of 2 mA/1.5 mA. *Tests were conducted in each group

The flowchart illustrating the process of the study is shown in Fig. 4. Seventy participants underwent the pre-enrollment screening evaluation. Of those, 10 did not meet inclusion criteria. From the 60 ultimately included patients, one participant from the 2 mA group and one participant from the sham group did not participate in the retention test. Age (2 mA group: 24.3 ± 2.7 years; 1.5 mA group: 24.6 ± 1.6 years; sham group: 25.3 ± 3.4 years) and sex (2 mA group: ten women; 1.5 mA group: 11 women; sham group: 11 women) did not differ between groups (*p* = 0.374 and *p* = 0.948, respectively). Mean values of movement time (s) and reaction time (s) by group and time are shown in Table 1. Movement time and reaction time did not differ between groups in the pretest (F(2,55) = 1.047, *p* = 0.358; and F(2,55) = 2.043, p = 0.139, respectively).

**Table 1:** Means, standard deviations and confidence intervals of movement time and reaction time for stimulation groups in time points

Variable	HD-tDCS 2 mA (n = 19)				HD-tDCS 1.5 mA (n = 20)				HD-tDCS sham (n = 19)			
	Pretest	During stimulation	Posttest	Retest	Pretest	During stimulation	Posttest	Retest	Pretest	During stimulation	Posttest	Retest
Movement time (s): Mean ± SD [*CI]	0.75 ± 0.17 [0.67–0.84]	0.77 ± 0.21 [0.67–0.87]	0.76 ± 0.22 [0.65–0.86]	0.73 ± 0.21 [0.63–0.83]	0.85 ± 0.26 [0.73–0.97]	0.81 ± 0.19 [0.72–0.90]	0.75 ± 0.17 [0.67–0.82]	0.73 ± 0.15 [0.66–0.80]	0.80 ± 0.20 [0.70–0.89]	0.81 ± 0.25 [0.69–0.93]	0.74 ± 0.21 [0.64–0.84]	0.70 ± 0.18 [0.62–0.79]
Reaction time (s): Mean ± SD [*CI]	0.34 ± 0.05 [0.32–0.37]	0.32 ± 0.05 [0.30–0.35]	0.32 ± 0.04 [0.30–0.34]	0.30 ± 0.03 [0.28–0.32]	0.31 ± 0.07 [0.27–0.34]	0.30 ± 0.04 [0.28–0.32]	0.30 ± 0.05 [0.27–0.32]	0.29 ± 0.05 [0.26–0.32]	0.34 ± 0.07 [0.31–0.38]	0.33 ± 0.03 [0.31–0.35]	0.32 ± 0.03 [0.31–0.34]	0.32 ± 0.04 [0.30–0.34]

*HD-tDCS 2 mA/1.5 mA* high-definition transcranial direct current stimulation with an intensity of 2 mA/1.5 mA. *CI = 95% confidence interval

### Effects on movement time (s)

A main effect of Time (F(3,165) = 15.969; *p* < 0.001; partial η2 = 0.23; observed power = 0.99) showed that across groups, movement time decreased significantly in both posttest (0.75 ± 0.20 s) and retest (0.72 ± 0.18 s) compared to the pretest (0.80 ± 0.22 s; vs. posttest: pBonferroni = 0.012; vs. retest pBonferroni < 0.001) and compared to during stimulation (0.80 ± 0.21 s; vs. posttest: pBonferroni < 0.001; vs. retest pBonferroni < 0.001). The interaction of Group x Time reached border-line significance (F(6,165) = 2.341, *p* = 0.034; corrected *p* (G-GE) = 0.060; partial η2 = 0.08; observed power = 0.80). Our interest was focused on clarifying whether movement time differed between groups at each time point and whether movement time differed between time points within each group. Therefore, despite the borderline significance of the corrected p value (G-GE), the interaction was further investigated.

Differences between groups at each time point: Movement time did not differ between groups at each timepoint (*p* ≥ 0.358).

**Fig. 5:**
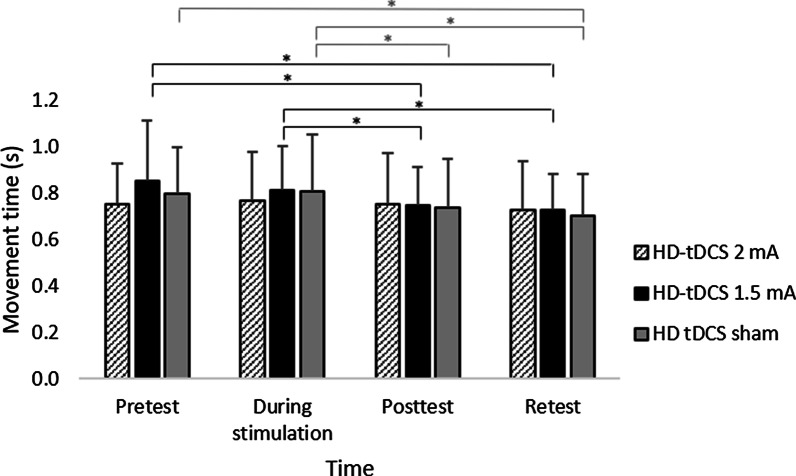
Mean movement time (s) of reaching movements in each group at the different time points. HD-tDCS 2 mA/1.5 mA = High-definition transcranial direct current stimulation with an intensity of 2 mA/1.5 mA. Error bars show standard deviation. Asterisks denote a significant difference (pBonferroni < 0.05). Black asterisks relate to the HD-tDCS 1.5 mA group and gray asterisks relate to the HD-tDCS sham group

Differences between time points within each group: Only for the 1.5 mA (F(3,57) = 11.707; *p* < 0.001; partial η2 = 0.38; observed power = 0.98) and the sham groups (F(3,54) = 9.593; *p* < 0.001; partial η2 = 0.348; observed power = 0.99), but not for the 2 mA group (F(3,54) = 0.945; *p* = 0.379; partial η2 = 0.05; observed power = 0.18), movement time decreased over time. In the 1.5 mA group, movement time decreased significantly in posttest (0.75 ± 0.17 s) and retest (0.73 ± 0.15 s) compared to pretest (0.85 ± 0.26 s, pBonferroni = 0.005 and pBonferroni = 0.015, respectively), and decreased significantly in posttest and retest compared to the movement time during the stimulation (0.81 ± 0.19 s, pBonferroni < 0.001 and pBonferroni = 0.003, respectively). In the sham group, movement time also decreased significantly in retest (0.70 ± 0.18 s) compared to pretest (0.80 ± 0.20 s, pBonferroni = 0.005), but as opposed to the 1.5 mA group, movement time in posttest did not differ from movement time in pretest (0.74 ± 0.21 s). Movement time also decreased significantly in posttest and retest compared to the movement time during stimulation (0.81 ± 0.25 s, pBonferroni = 0.002 and pBonferroni = 0.004, respectively) (Fig. 5).

**Fig. 6:**
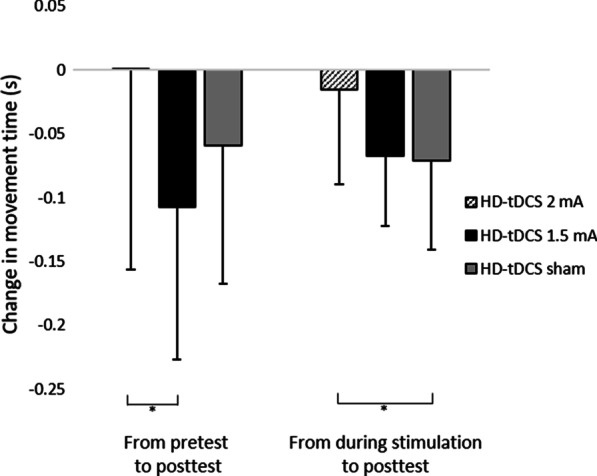
Change in movement time (s) of reaching movements from pretest to posttest and from the course of stimulation to posttest in each group. HD-tDCS 2 mA/1.5 mA = High-definition transcranial direct current stimulation with an intensity of 2 mA/1.5 mA. Error bars show standard deviation. Asterisks denote a significant difference (pBonferroni < 0.05)

**Fig. 7:**
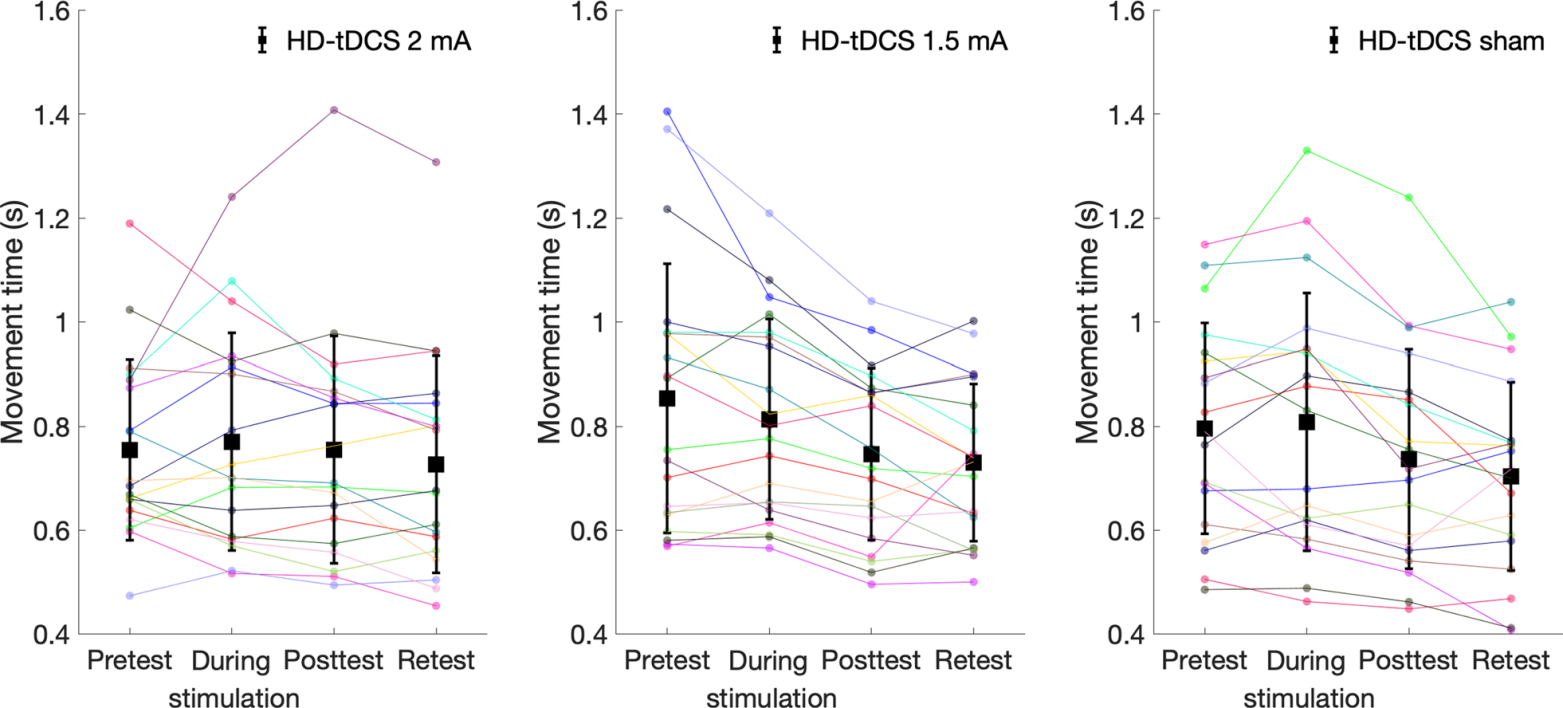
Individual movement time (s) in each group at the different time points. HD-tDCS 2 mA/1.5 mA = High-definition transcranial direct current stimulation with an intensity of 2 mA/1.5 mA. Black squares show mean movement time, and error bars show standard deviation

Differences in change in movement time between groups: Change in movement time from pretest to posttest and from the course of stimulation to posttest differed significantly between groups (F(2,55) = 3.358, p = 0.042; F(2,55) = 4.166, *p* = 0.021, respectively). In the 1.5 mA group, reduction in movement time from pretest to posttest (change value: -0.11 ± 0.12 s) was significantly greater compared to the 2 mA group (change value: 0.00 ± 0.16 s, pBonferroni = 0.037). In the sham group, reduction in movement time from the course stimulation to posttest (change value: -0.07 ± 0.07 s) was significantly greater compared to the 2 mA group (pBonferroni = 0.038) (Fig. 6). Indeed, as can be seen in Fig. 7, movement time deteriorated (increased) from pretest to posttest in two subjects in the 1.5 mA group and in five in the 2 mA group, and movement time deteriorated (increased) from course stimulation to posttest in two subjects from the sham group and in five from the 2 mA group. No other significant effects were observed. Additional file 2: Figure S1 and Additional file 3: Figure S2 show the mean movement time and reaction time within each sequence for each of the two blocks at each time point (pretest, during stimulation, posttest, retention test) for each group.

### Effects on reaction time (s)

**Fig. 8:**
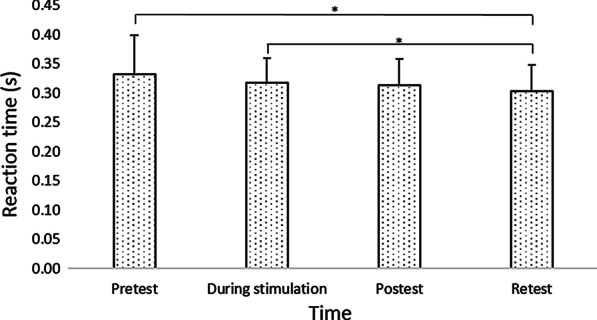
Mean reaction time (s) of reaching movements at the different time points. HD-tDCS 2 mA/1.5 mA = High-definition transcranial direct current stimulation with an intensity of 2 mA/1.5 mA. Error bars show standard deviation. Asterisks denote a significant difference (pBonferroni < 0.05). Main effect of Time (collapsed across the groups) is presented because the interaction Group x Time was not significant

A main effect of Time (F(3,165) = 9.473; p < 0.001; partial η2 = 0.15; observed power = 0.98) showed that across groups, reaction time decreased significantly in retest (0.30 ± 0.04 s) compared to pretest (0.33 ± 0.07 s; pBonferroni < 0.001) and during stimulation (0.32 ± 0.04 s; pBonferroni = 0.009) (Fig. 8). No other significant effects were observed.

### Adverse effects

The stimulation was well tolerated by the participants, and no sessions were aborted due to adverse effects. The occurrence of adverse effects in the 2 mA; 1.5 mA and sham groups are displayed in Table 2. Frequency of adverse effects did not differ between the groups. Strength of the discomfort from the adverse effects differed between groups (p < 0.001) such that it was significantly higher in the 2 mA (median: 3, interquartile range: 2–4) and 1.5 mA (median: 4.5, interquartile range: 3.25–5) groups compared to the sham group (median: 1, interquartile range: 0–3; p < 0.001 for all), but there was no difference between the 2 mA and 1.5 mA groups.

**Table 2:** Frequency of adverse effect

Symptom	HD-tDCS 2 mA (n = 19)	HD-tDCS 1.5 mA (n = 20)	HD-tDCS sham (n = 19)
Tingling	17 (90%)	12 (60%)	15 (79%)
Burning sensation	6 (32%)	9 (45%)	8 (42%)
Itching	4 (21%)	9 (45%)	6 (32%)
Hair pulling	1 (5%)		1 (5%)
Irritating		1 (5%)	1 (5%)

*HD-tDCS 2 mA/1.5 mA* high-definition transcranial direct current stimulation with an intensity of 2 mA/1.5 mA

## Discussion

To the best of our knowledge, this is the first study to evaluate the effects of stimulation intensity on motor performance using HD-tDCS applied with optimized electrode configurations for maximal focal anodal stimulation of the primary motor cortex in healthy subjects. By applying such focal stimulation, in contrast to conventional tDCS with large sponge electrodes, a more nuanced determination of current intensity effects related to the stimulation of the primary motor cortex was achieved. The present study showed that at each time point, the movement time of a sequence of reaching movements did not differ between 2 mA, 1.5 mA and sham groups in healthy subjects following a single 20-min session of HD-tDCS over M1 contralateral to the left, non-dominant hand performing the motor task. However, it was only following HD-tDCS at 1.5 mA and sham HD-tDCS that movement time improved at retest as compared to pretest, and at posttest and retest as compared to movement time during stimulation. An immediate effect at posttest, as compared to pretest, was only found after the 1.5 mA HD-tDCS. This reduction in movement time from pretest to posttest was significantly greater compared to HD-tDCS 2 mA. In HD-tDCS at 2 mA, the negligible reduction in movement time from the course of stimulation to posttest was significantly lower compared to sham HD-tDCS. Reaction time improved in a similar manner following 1.5 mA, 2 mA, and sham stimulations in retest as compared to pretest and to the reaction time during the stimulation.

The improvement found after sham stimulation reflects motor learning over time, and potentially also a placebo effect. The motor learning over time can be related to the initial fast-learning phase within session and a slow, across-session phase due to consolidation [41, 42]. The placebo effect can be related to the finding that the frequency of adverse effects following two min of stimulation did not differ between the groups (though the strength of the discomfort from the adverse effects was significantly higher in the 2 and 1.5 mA groups compared to the sham group). Similarly, previous studies found that subjects were not able to distinguish between active and sham tDCS [43, 44].

However, movement time improved at posttest as compared to pretest solely after HD-tDCS at 1.5 mA. This improvement in motor performance is only partly in line with our hypothesis that 1.5 mA HD-tDCS would be more effective in decreasing movement time than sham tDCS as this improvement was found within the 1.5 mA group, but movement time in posttest did not differ between 1.5 mA and sham groups. This improvement is in line with some previous findings that conventional atDCS [45–48] and the recently developed HD-tDCS [31, 32] to the primary motor cortex can facilitate motor performance and learning in healthy subjects. The inconsistency between our findings and those of Cyprus et al. [21]–who also compared the effects of tDCS intensities on motor performance and found a significant difference of motor performance in an 8-element finger sequence for atDCS with large sponge electrodes at 1.5 mA as compared to sham tDCS using a crossover design–may relate to the different study design, motor tasks, and/or tDCS montages (in both studies, a single session of 20 min was conducted).

Whereas movement time improved following HD-tDCS at 1.5 mA and sham tDCS, it did not improve following HD-tDCS at 2 mA. Movement time did not differ between the groups at baseline, yet only the HD-tDCS at 2 mA did not elicit improvement over time. These findings are partly in line with our hypothesis that 1.5 mA HD-tDCS would be more effective in decreasing movement time than 2 mA because the specific improvements were found in the 1.5 mA, although movement time in posttest/retest did not differ between these groups. Findings of previous studies that investigated the effects of current intensity on neurophysiological [2, 9–18] and behavioral [20–24] measures are inconsistent. It should be noted, however, that comparing studies that investigated the effect of current intensity on neurophysiological and behavioral measures may be too simplistic, because the complete dose of tDCS is defined not only by the applied current, but also by the current duration and electrode montage, all of which produce a complex pattern of current flow in the brain [9, 49]. In addition, the amount of current density to the brain may vary for the same applied current due to individual anatomical differences, which may, therefore, lead to variations in individual intensity-response [50].

With respect to the findings that movement time did not differ between time points following HD-tDCS at 2 mA for 20 min, and that the negligible reduction in movement time from the course of stimulation to posttest was even smaller in the 2 mA group as compared to the sham group, it appears that this current intensity in this particular experimental setup probably inhibited the learning effects. Indeed, individual inspection of the percentage of deterioration between two time points (from pretest to posttest/course of stimulation/retest and from during the course of stimulation to posttest/retest), shows that deterioration was highest in the 2 mA group (32%), followed by the sham (27%) and 1.5 mA (16%) groups (Fig. 7).

The interpretation regarding inhibited learning effects is based on previous findings, which indicated that increasing current intensity may switch the direction of MEP after-effects [13, 18, 19, 51]. Batsikadze et al. [13] found that conventional cathodal tDCS, applied with M1-contralateral orbit montage at 2 mA intensity for 20 min, led to excitability rather than inhibition, as measured by MEP changes. Shilo and Lavidor [52], using a reaction time task, found that conventional anodal stimulation at 2 mA led to faster performance than cathodal stimulation at 2 mA, but only before 13 min of stimulation had elapsed; after 13 min, the pattern switched, and performance under cathodal stimulation was faster. They concluded that cathodal tDCS has a non-linear effect, and that the known polarity-dependent effects of cathodal tDCS shift after 13 min of stimulation, leading to increased, rather than decreased excitability. The switching pattern of MEP amplitudes was also found after 26 min of conventional atDCS of the left primary motor cortex at lower current intensity—1 mA [51]. However, when two 13 min blocks of atDCS were separated by 20 min, after-effects were present for up to 24 h, suggesting the involvement of late-phase, long-term potentiation plasticity [51]. It has been suggested that the change of cortical excitability from excitation to inhibition is related to neuronal inhibitory mechanisms that have a delayed onset when exposed to excitatory protocols [53]. Recent results suggest a calcium-dependency of the directionality of tDCS-induced neuroplasticity [54]. The aforementioned findings, along with those of our study, suggest that the classic excitatory and inhibitory effects do not hold for every stimulation protocol. This suggestion may at least partially explain the inconsistent previous findings of the effects of tDCS on motor behavior using variable stimulation protocols [55–61]. Switching neurophysiological and behavioral patterns following prolonged stimulation may result in better motor performance following combined partly anodal and partly cathodal stimulation; however, this requires further investigation.

A similar effect on reaction time was found following 1.5 mA, 2 mA, and sham stimulations. Reaction time reflects motor preparation, whereas movement time characterizes movement execution. Indeed, a recent meta-analysis [5] demonstrated a modest improvement in reaction time with an effect size smaller than that of the execution time following tDCS in healthy participants [26]. The primary motor cortex is more closely related to the execution of the selected response [62–64], whereas the premotor cortex is more involved in the selection and preparation of motor responses. It is likely that reaction time improved across groups due to training, as is common in similar sequence learning tasks [65]. In contrast, the execution task in the current study was to perform a sequential point-to-point movement task with the non-dominant hand toward small, 0.5 cm diameter targets on the graphics tablet. Due to the difficulty of the execution task and the amount of room for improvement, this measure may have been more affected by stimulation intensity. Another possible explanation for the differential effect of stimulation intensity on reaction time vs. movement time may relate to the montage used. In the current study, the primary motor cortex was targeted using HD-tDCS with optimized electrode configurations for maximal focal stimulation. Even so, the current flow also reached the premotor cortex, as Fig. 2 indicates. It is yet to be determined if targeting adjacent areas–the premotor cortex vs. primary motor cortex–using HD-tDCS with optimized electrode configurations for maximal focal stimulation (based on current-flow models), can differentially affect reaction time vs. movement time.

Several caveats of the current study need to be taken into consideration. First, despite using HD-tDCS, which reduces the current spread and targets the primary motor cortex more focally than conventional large pad tDCS [9], the electric fields (V/m) could have differed between groups because of the individual subjects’ anatomical features [50]. Variations in the electric fields are mainly caused by differences in the individual morphology of the cerebrospinal fluid and brain, and hence, cannot be controlled in experimental studies unless detailed image processing is performed. Indeed, many studies have shown that 20–60% of a group of individuals experience the classical excitability increase induced by a single atDCS session [10, 14, 66–71]. Also, individual tDCS-induced plasticity in primary motor cortex, as indexed by alterations in GABA following atDCS, is related to individual motor learning capacity [72, 73]. Second, applying frequent behavioral and electrophysiological (MEPs) measurements during the stimulation could have helped determine if and when the polarity shifted during the HD-tDCS at 2 mA. Third, it is possible that a more challenging task with more room for improvement in motor performance of healthy participants would have emphasized the differences between groups. For example, randomizing the holding time of the stylus at the starting point, instead of keeping it constant for 500 ms, could have increased the possibility of improvement in reaction time in the current explicit learning task. Fourth, although the frequency of adverse effects following two min of stimulation did not differ between the groups, the strength of the discomfort from the adverse effects was significantly higher in the 2 and 1.5 mA groups compared to the sham group. It could have affected the subjects` blinding. It should be noted, though, that the differences in movement time between the 2 and 1.5 mA groups were not influenced from adverse effects as both the frequency and strength of the discomfort did not differ between the groups. Nonetheless, whereas the cutaneous sensations associated with the sham stimulation contribute to blinding, they may also create methodological implications, as was recently suggested by van Boekholdt et al. [74], who indicated that tDCS could also have an effect through a peripheral route. Action potentials in peripheral nerves underlying tDCS electrodes can be initiated due to the high electric field strengths [74–76]. Consequently, the somatosensory system is activated [77]. Their hypothesis is that tDCS induces arousal and vigilance through the peripheral mechanisms that involve peripherally evoked activation of the ascending reticular activating system, in which norepinephrine is distributed throughout the brain by the locus coeruleus. The standard tDCS sham condition, as in this study, does not control for this transcutaneous route. This may have, in part, influenced the improvement in motor performance measured after the sham stimulation.

## Conclusion

In the current montage of 20-min anodal HD-tDCS with maximal focal stimulation of the primary motor cortex, movement time and reaction time did not differ between groups in each timepoint. However, only 1.5 mA was effective for inducing immediate improvement of movement time at posttest as compared to pretest, and 2 mA reduced learning and placebo effects. These findings suggest that excitatory effects induced by anodal stimulation do not hold for every stimulation intensity, and may help in the development of optimal HD-tDCS-based therapeutic protocols aimed at improving upper limb functioning in people with stroke. Given the complex interactions between lesion characteristics, spontaneous recovery, and training in people with stroke, determining the best way to optimize stimulation protocols for enhancing tDCS effects on motor performance is important to be accomplished by means of challenging motor tasks in healthy subjects. Further systematic investigation of the optimal current intensity, using a wider range of intensities, for enhancing behavioral responses is needed to improve the translation of tDCS from the realm of research to clinical practice.

## Supplementary Information

**Additional file 1: Table S1.** Characteristics of studies that investigated the effects of transcranial direct current stimulation intensity on neurophysiological and behavioral measures.

**Additional file 2: Fig. S1.** Mean movement time (s) of reaching movements at the blocks in the different time points in the groups. 2 mA/1.5 mA = High-definition transcranial direct current stimulation with an intensity of 2 mA/1.5 mA. Error bars show standard error of the mean.

**Additional file 3: Fig. S2.** Mean reaction time (s) of reaching movements at the blocks in the different time points in the groups. 2 mA/1.5 mA = High-definition transcranial direct current stimulation with an intensity of 2 mA/1.5 mA. Error bars show standard error of the mean.

## Data Availability

The data will be made available from the authors based on reasonable requests.

## Abbreviations

atDCS: Anodal transcranial direct current stimulation
ANOVA: Analysis of variance
HD: High-definition
MEP: Motor evoked potentials
M1: Primary motor cortex
