# Supplementary material for: The effect of high-definition transcranial direct current stimulation intensity on motor performance in healthy adults: a randomized controlled trial
Source: J Neuroeng Rehabil. 2021 Jun 26;18:103. doi: 10.1186/s12984-021-00899-z (PMC8236155; doi:10.1186/s12984-021-00899-z)
Supplement: Supplementary file 1 — Additional file 1: Table S1. Characteristics of studies that investigated the effects of transcranial direct current stimulation intensity on neurophysiological and behavioral measures. [file 12984_2021_899_MOESM1_ESM.docx]

**Supplementary table 1**

Title: Characteristics of studies that investigated the effects of transcranial direct current stimulation intensity on neurophysiological and behavioral measures.

| References | Sample  size | Intervention | tDCS site,  reference site | Current (mA) | Duration (min) | Results |
| --- | --- | --- | --- | --- | --- | --- |
| Neurophysiological studies | | | | | | |
| Nitche and Paulus, 2000 [1] | 12 | atDCS | L M1,  C-SOR | 0.2, 0.4, 0.6, 0.8, 1 | 5 | Increasing intensity led to prolonged and larger after ­effects in MEPs. |
| Ammann et al., 2017 [10] | 12 | atDCS | L M1,  C-SOR | 1 vs. 2 | 7 | 2 mA and not 1 mA significantly increased MEP. 2 mA anodal tDCS resulted in consistent intra- and inter-individual increases of MEPs. |
| Bastani and Jaberzadeh, 2013 [11] | 12 | atDCS | L M1,  C-SOR | 0.3, 0.7, 1.4, 2 | 10 | 0.3 mA induced significantly larger MEP than 0.7 mA. No significant differences between 0.3 mA and 1.4 mA or 0.3 mA and 2 mA. |
| Agboada et al., 2019 [12] | 16 | atDCS | L M1,  C-SOR | 1, 2, 3 | 15-30 | All active stimulation conditions enhanced MEPs. No significant differences in MEPs between stimulation intensities and durations.  A trend for larger MEPs for higher current intensities (1 vs. 3mA). |
| Chew et al., 2015 [14] | 29 | atDCS | L M1,  C-SOR | 0.2, 0.5, 1, 2 | 10 | No differences in MEPs between the four current intensities. |
| Ho et al., 2016 [15] | *89 | atDCS | L M1,  C-SOR | 1, 2 | 10, 20 | No differences in MEPs between the current intensities. |
| Jamil et al., 2017 [16] | 38 | atDCS and ctDCS | L M1,  C-SOR | 0.5, 1, 1.5, 2 | 15 | For both tDCS polarities, the MEPs did not linearly correlate with increasing intensity; effects of lower intensities (0.5, 1.0 mA) showed equal, if not greater MEPs. |
| Kidgell et al., 2013 [17] | 14 | atDCS | L M1,  C-SOR | 0.8, 1, 1.2 | 10 | No differences in MEPs between the current intensities. |
| Batsikadze et al., 2013 [13] | 14 | atDCS and ctDCS | L M1,  C-SOR | 1, 2 | 20 | atDCS and ctDCS at 2 mA resulted in increased MEP, whereas 1 mA cathodal tDCS resulted in decreased MEP. |
| Moliadze et al., 2015 [18] | 29 | atDCS and ctDCS | L M1,  C-SOR | 0.5, 1 | 10 | MEPs increased after both 1 mA cathodal and anodal stimulation. The 0.5 mA cathodal stimulation caused a significant decrease of MEP, whereas the 0.5 mA anodal stimulation had no effect at all. |
| Strube et al., 2016 [19] | 59 | atDCS and  ctDCS | L M1,  C-SOR | 1 | 13 (atDCS) and 9 (ctDCS) | atDCS resulted in increased MEP, whereas cathodal tDCS did not alter corticospinal excitability. |
| Behavioral studies | | | | | | |
| Boggio et al., 2006 [20] | 18 | atDCS | L M1 and  L DLPFC,  C-SOR | 1, 2 | 20 | A significant improvement in working memory of patients with PD after active atDCS of the LDLPFC with 2 mA. Treatment with 2 mA induced a differential, larger effect compared with 1 mA. |
| Iyer et al., 2005 [22] | 103 | atDCS and ctDCS | L PFC,  C-SOR | 1, 2 | 20 | No significant effects on performance with 1 mA tDCS. At 2 mA, verbal fluency improved significantly with anodal and decreased mildly with ctDCS. |
| Shekhawat et al., 2016 [24] | 27 | aHD-tDCS | L TA and DLPFC,  R for LTA: 4 adjoining cathodes at C5, TP7, CP3, and P5 and R For DLPFC: 4 adjoining cathodes at F2, FC4, F6, and AF4 | 1, 2 | 10, 20 | Higher intensity (2 mA) and longer duration (20 minutes) of stimulation resulted in greater reduction in both tinnitus loudness and annoyance than 1 mA in participants with chronic tinnitus. |
| Cuypers et al., 2013 [21] | 13 | atDCS | M1 contralateral to the dominant hand,  C-SOR | 1, 1.5 | 20 | No differences in motor performance of finger sequence between 1.5 mA atDCS and 1 mA atDCS; and between 1 mA atDCS and sham tDCS.  A significant increase in the slope of the learning curve and a significant improvement in motor performance at retention for 1.5 mA atDCS as compared to sham tDCS. |
| Horvath et al., 2016 [26] | 150 | AtDCS and ctDCS | L M1,  C-SOR | 1, 2 | 20 | No significant effect of tDCS on measures of simple visual motor reaction time following 1 mA and 2 mA atDCS or cathodal tDCS. |
| Mitroi et al., 2020 [23] | 102 | atDCS and ctDCS | L Cerebellum,  Ipsilateral buccinator muscle | 1.5, 2 | 20 | tDCS facilitated cerebellar-dependent timing of delay eyeblink conditioning, irrespective of stimulation intensity and polarity. |
| Ehrhardt et al., 2021 [25] | 123 | atDCS | L PFC,  C-SOR | 0.7, 1, 2 | 20 (four sessions) | Both 0.7 mA and 1 mA influenced some aspects of task performance, whereas 2.0 mA did not. 1 mA stimulation augmented cognitive performance compared with sham. Training gains transferred to an untrained response-selection paradigm only following 1 mA. |

C-SOR = Contralateral Supraorbital Region; DLPFC = dorsolateral prefrontal cortex; HD = high definition; L = left; MEP = Motor Evoked Potentials; PD = Parkinson’s disease; PFC = prefrontal cortex; R = reference site; TA = temporoparietal area; tDCS = transcranial direct current stimulation; a/ctDCS = anodal/cathodal tDCS. If not otherwise noted, the studies were conducted with healthy subjects using tDCS with sponge electrodes. *Pooled data from seven studies that were conducted by the same research team at the same research centre using the same equipment and experimental procedure.
