# Supplementary figures and images for: The effect of high-definition transcranial direct current stimulation intensity on motor performance in healthy adults: a randomized controlled trial
Source: J Neuroeng Rehabil. 2021 Jun 26;18:103. doi: 10.1186/s12984-021-00899-z (PMC8236155; doi:10.1186/s12984-021-00899-z)

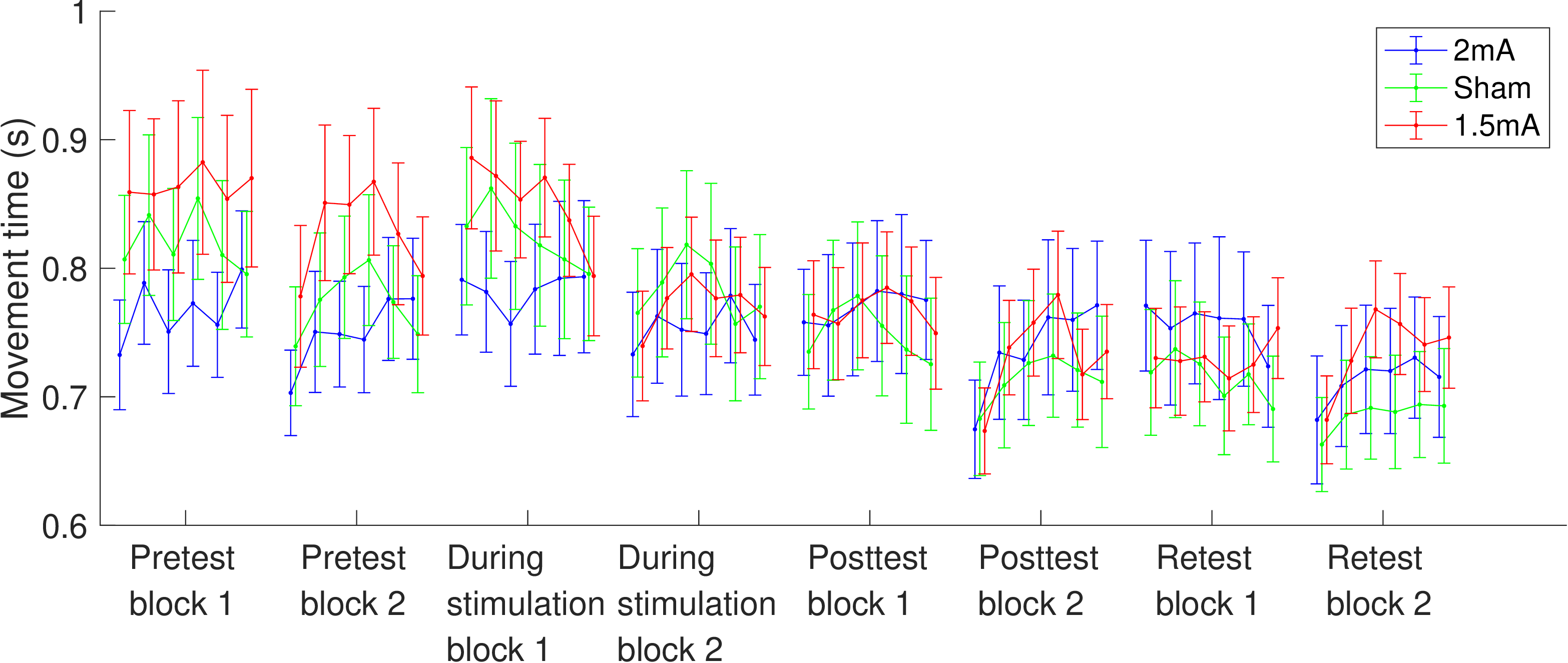

Supplement: Supplementary file 2 — Additional file 2: Fig. S1. Mean movement time (s) of reaching movements at the blocks in the different time points in the groups. 2 mA/1.5 mA = High-definition transcranial direct current stimulation with an intensity of 2 mA/1.5 mA. Error bars show standard error of the mean. [file 12984_2021_899_MOESM2_ESM.tif]

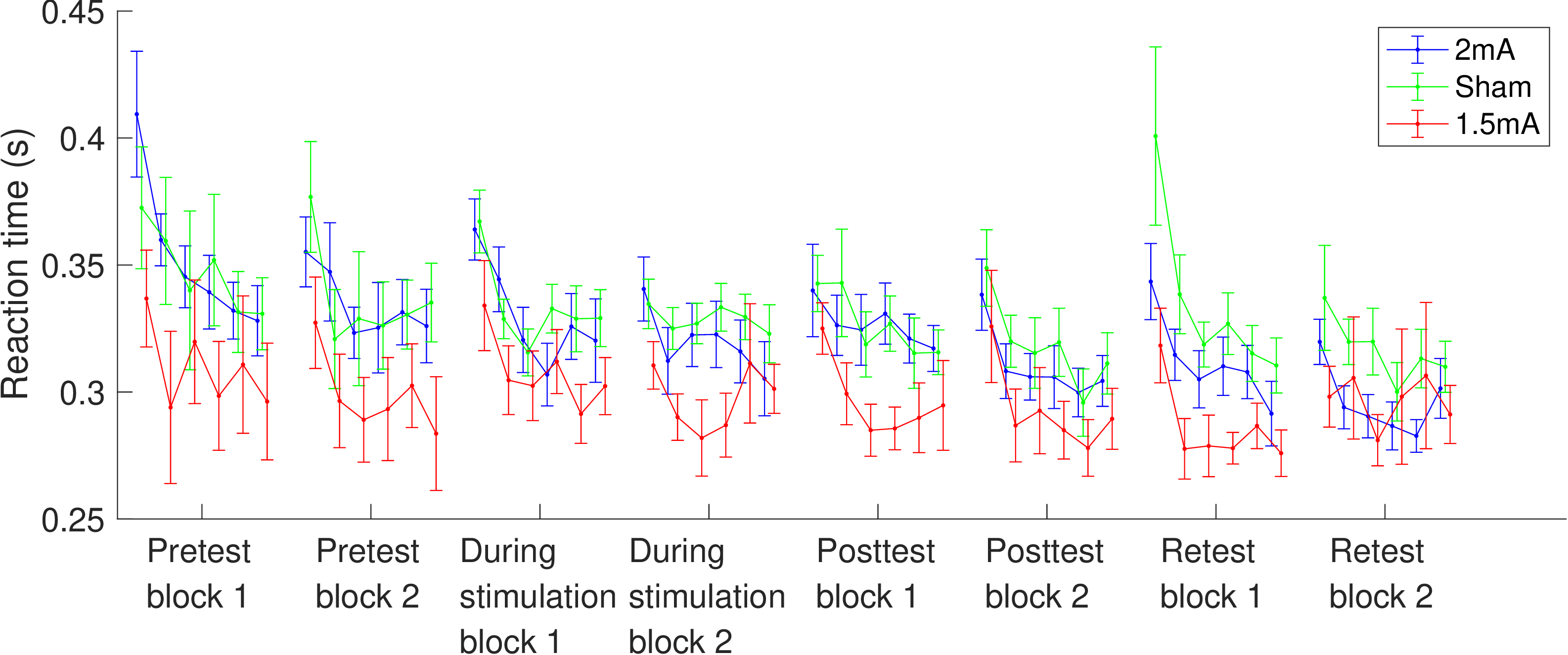

Supplement: Supplementary file 3 — Additional file 3: Fig. S2. Mean reaction time (s) of reaching movements at the blocks in the different time points in the groups. 2 mA/1.5 mA = High-definition transcranial direct current stimulation with an intensity of 2 mA/1.5 mA. Error bars show standard error of the mean. [file 12984_2021_899_MOESM3_ESM.tif]
